# Comment on “Graft Inflow Modulation by Splenic Artery Ligation for Portal Hyper Perfusion Does Not Decrease Rates of Early Allograft Dysfunction in Adult Live Donor Liver Transplantation: A Randomized Control Trial”: Can We Really Write Off Graft Inflow Modulation

**DOI:** 10.1097/AS9.0000000000000474

**Published:** 2024-07-16

**Authors:** Niteen Kumar, Abhideep Chaudhary

**Affiliations:** From the * Department of HPB Surgery and Liver Transplantation, Institute For Digestive & Liver Diseases, B L Kapoor-Max Super Speciality Hospital, New Delhi, India.

We read the article by Pamecha et al^1^ with great interest and congratulate the authors for this endeavor. However, there are important observations we would like to make. As the study shows, there is a significant fall in portal venous pressure (PVP, mm Hg) from 20.2 ± 3.6 to 16.4 ± 2.3, portal venous flow (PVF, mL/min/100 g) from 377.7 ± 82.6 to 269.3 ± 61.5, and PVP-central venous pressure from 10.5 ± 3.4 to 6.8 ± 2.9. It is worth noting that, despite this fall in pressure and flow, it is still in the range of “high” PVP and PVF. Though the graft inflow modulation (GIM) essentially reduces pressure and flows, it seems inadequate. In simple words, hyperperfusion persists. This is further clear from the Figure 2A, B. The actual fall in PVP to 15 mm Hg or less happens in only 13.15% (5/38) patients and among these 2 had PVP of 15 mm Hg or less to begin with. Similarly, PVF fell to <250 mL/min/100 g in no more than 23% (9/38) of the patients with at least 4 patients having PVF <250 mL/min/100 g to begin with. One could argue if hyperperfusion is persisting despite GIM, early allograft dysfunction is unlikely to improve. This probably explains the fact that despite having a “statistically significant” fall in PVP and PVF (in the GIM arm), a “clinically significant” benefit was not seen in this randomized controlled trial. Authors have contended that multiple factors play a role^2^ in patients developing early allograft dysfunction. Portal hyperperfusion per se is not the only factor. In our view, that is partially true as far as the present study is concerned. First, being a randomized controlled trial, both the groups must have experienced the similar effect of “other” factors, and as the intervention was limited to SAL, it is nonetheless affecting only portal hemodynamic. Second, most of these factors can be easily controlled in a living donor liver transplant setting. As the authors have excluded deceased donor liver transplant from the study, there is no way of knowing their impact in this situation.
